# Equisetin Targets Intracellular *Staphylococcus aureus* through a Host Acting Strategy

**DOI:** 10.3390/md20110656

**Published:** 2022-10-22

**Authors:** Jiayao Tian, Shang Chen, Fei Liu, Qian Zhu, Jianzhong Shen, Wenhan Lin, Kui Zhu

**Affiliations:** 1 National Center for Veterinary Drug Safety Evaluation, College of Veterinary Medicine, China Agricultural University, No. 2 Yuanmingyuan West Road, Beijing 100193, China; 2 Guangdong Laboratory for Lingnan Modern Agriculture, Guangzhou 510642, China; 3 State Key Laboratory of Natural and Biomimetic Drugs, Peking University, Beijing 100191, China

**Keywords:** marine drug, equisetin, host-acting compound, autophagy

## Abstract

Mammalian cells act as reservoirs of internalized bacteria to circumvent extracellular antibacterial compounds, resulting in relapse and reinfection diseases. The intracellular persistence of *Staphylococcus aureus* renders most traditional antibiotics useless, due to their inadequate subcellular accumulation. To replenish our antibiotic arsenal, we found that a marine-derived compound, equisetin, efficiently eliminates intracellular *S. aureus* by potentiating the host autophagy and inducing mitochondrial-mediated ROS generation to clear the invading *S. aureus*. The remarkable anti-infection activity of equisetin was validated in a peritonitis-infected mouse model. The marine product equisetin utilizes a unique dual mechanism to modulate the host–pathogen interaction in the clearance of intracellular bacteria. Thus, equisetin is an inspiring host-acting candidate for overcoming intracellular pathogens.

## 1. Introduction

Apart from classical intracellular bacteria like *Listeria monocytogenes*, *Staphylococcus aureus* previously regarding as extracellular bacteria, has been reported to create a protective niche inside the professional phagocytes, such as macrophages and neutrophils, and non-professional phagocytes including endothelial cells and epithelial cells [1]. The cumulative evidence shows that *S. aureus* is able to invade and replicate in host cells, inducing cell death to escape and start a new cycle of infection, and ultimately resulting in various latent or recurrent diseases [2,3,4,5]. Compared to the passive endocytosis of the professional phagocytes, *S. aureus,* equipped with the staphylococcal surface protein fibronectin binding proteins (FnBP) A and B, employs a zipper-type mechanism to invade non-professional phagocytes [6]. The internalized *S. aureus* secretes the pore-forming toxin, alpha-hemolysin, to activate autophagy and is transited into the autophagosome for degradation [7]. The *S. aureus* employs sophisticated strategies to inhibit autophagosome maturation and fusion with lysosome and survives inside host cells [8].

Within the autophagosome, the *S. aureus* is protected from extracellular antibacterial compounds. Classic antibiotics, such as β-lactams and aminoglycosides, are restricted from penetrating mammalian cells due to their high hydrophilicity, whereas other antibiotics, such as fluoroquinolones and macrolides, are permeable but inadequately accumulate in host cells. This subtherapeutic accumulation of antibiotics inside the cells may contribute to the development of antibiotic tolerance in *S. aureus* [9]. To date, it urgently calls for the development of new strategies to combat intracellular bacterial infections [10,11,12]. Previous studies have highlighted that host-acting compounds potentiate the host defense, thus providing a promising approach to diminish internalized bacteria [13]. For instance, aggregation-induced emission luminogens TBP-1, activate the host autophagy to clear internalized *S. aureus* [14]. Notably, anti-mycobacterial drug bedaquiline triggers the host phagosome fusion and autophagy to reprogram cells into potent bactericidal phagocytes [15]. Importantly, host-acting agents are less likely to contribute to bacterial resistance, as they exert antibacterial activities by boosting the inherent host defense.

To expand the antibiotic arsenal, we isolated a promising antibacterial compound, equisetin, from a sponge-associated fungus, *Fusarium equiseti* 33-10 [16]. Equisetin is an N-methylserine-derived acyl tetramic acid, first isolated from the deep-sea fungus *Fusarium equiseti* NRRL 5337 [17,18]. It exerts a robust antibacterial activity against MDR bacteria, especially Gram-positive pathogens, by a novel mode of action involving allosterically binding to biotin carboxylase [19,20]. In addition, equisetin also inhibits HIV-Ι integration and the quorum-sensing in *P. aeruginosa* [21,22]. However, most efforts have been focuse on the interaction between such compound and pathogens, but quite limited options have been developed from the perspective of hosts. Here, we found that equisetin shows an intracellular bacteria elimination ability in an *S. aureus*-infected cell model. As mentioned above, ablating intracellular bacteria by host-acting agents is safer and less likely to increase resistance than traditional drugs. Thus, we hypothesize that equisetin is a host-acting candidate against intracellular bacteria.

## 2. Results

### 2.1. Equisetin Exerts Intracellular Bactericidal Ability through Host-Acting Interaction

**Figure 1:**
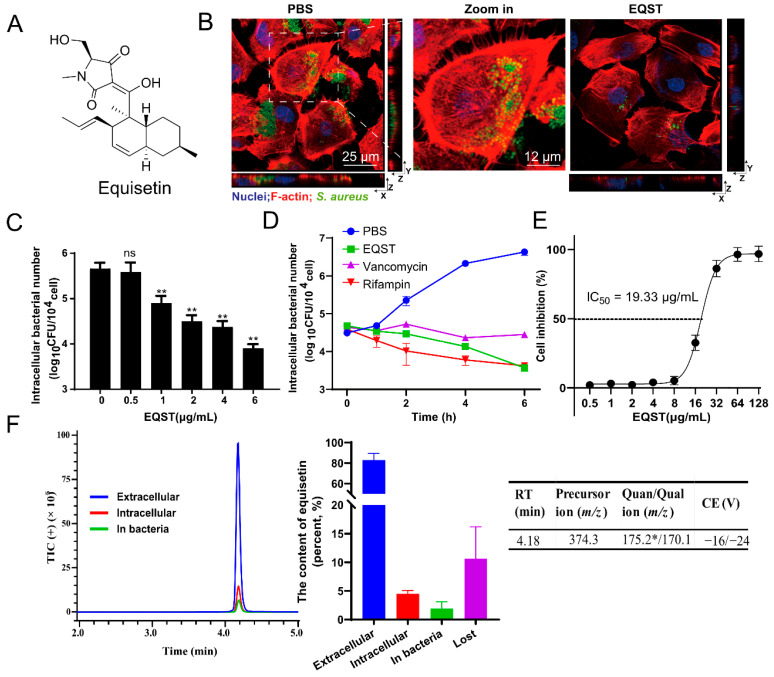
Equisetin (EQST) kills intracellular *S. aureus* by targeting host cells. (**A**) The chemical structure of equisetin. (**B**) The representative images of intracellular *S. aureus* ATCC 29213 in IEC-6 cells treated with PBS or 4 μg/mL equisetin (MOI = 1, infected 4 h). Extracellular *S. aureus* was removed by the treatment with gentamicin at 100 μg/mL for 30 min. (**C**) The number of intracellular *S. aureus* in IEC-6 cells treated with equisetin (0–6 μg/mL) (MOI = 1, infected 4 h). (**D**) The number of intracellular *S. aureus* in IEC-6 cells treated with 4 μg/mL equisetin at different time pionts. (**E**) Cytotoxicity of IEC-6 cells in the absence or presence of equisetin for 24 h. Cytotoxicity is presented as cell inhibition percentage. ((**F**), left and middle) Distribution of extracellular antibiotics, intracellular antibiotics in the cytosol of IEC-6 cells, and antibiotics in *S. aureus* quantified by LC–MS/MS. The loss of antibiotics was calculated from the total antibiotics minus the other detectable antibiotics. ((**F**), right) Basic information of LC/MS method. RT, retention time. *: quantitative ion. Data are representative of three independent experiments and values are expressed in mean ± SD. ** *p* < 0.01, ns p > 0.05, *p*-values were determined by unpaired *t*-tests, using PRISM 8.0.

To examine the activity of equisetin against intracellular bacteria, we used rat intestinal epithelioid cells (IEC-6) infected with *S. aureus* as a model, according to our previous work [14]. We initially assayed the antibacterial ability of equisetin against *S. aureus* ATCC 29213, internalized within IEC-6 cells (Figure 1A). The internalized bacteria were effectively eliminated after equisetin treatment (Figure 1B). Further, we assessed the survival of intracellular bacteria at different concentrations of equisetin and found that 4 μg/mL of equisetin effectively killed bacteria after the treatment for 4h(Figure 1C). This concentration did not affect the cell viability over an incubation period of 24 h (Figure 1E). In addition, equisetin decrease the number of internalized bacteria in a time-dependent manner and presented a better intracellular bacterial clearance characteristic than vancomycin (Figure 1D). To evaluate the cytotoxicity of equisetin to mammalian cells, we performed a WST-1 cell viability assay and found that equisetin minimally intoxicated IEC-6 cells at 0–8 μg/mL (IC_50_ = 19.33 μg/mL), suggesting that the reduction of intracellular bacteria was not related to the cell death (Figure 1E). Compared with the antibiotics used in clinic against *S. aureus* associated infections [23], equisetin showed a more efficacious intracellular activity.

Since equisetin displayed potent antibacterial ability against *S. aureus* in the broth culture medium, we presumed that equisetin may accumulate in the host cell to eradicate internalized *S. aureus*. To quantify the distribution of equisetin in the infection cell model, we analyzed its content via mass spectrometric (liquid chromatography—tandem mass spectrometry (LC-MS/MS)) analysis and found only approximately 5% equisetin (4 μg/mL) accumulated in IEC-6 cells (Figure 1F). Since the drug concentration in the subcellular location was far below an effective concentration, the vast majority of equisetin that exists outside IEC-6 cells playing a dominant role in the intracellular bactericidal process. Therefore, equisetin may hijack an unique mechanism that is distinct from the direct antibacterial activity. In addition, a higher penetration of antibiotics is often accompanied with a higher cytotoxicity to mammalian cells. The low cytotoxicity of equisetin shows its potential as a new antibiotic, without destroying the mammalian cells (Figure 1D). Despite its low cellular accumulation, equisetin still displays potent intracellular ability; thus, we hypothesized that equisetin eliminates the internalized *S. aureus* in a host-acting way.

### 2.2. The Internalized Bactericidal Activity of Equisetin Is Attributed to Autophagy Activation

**Figure 2:**
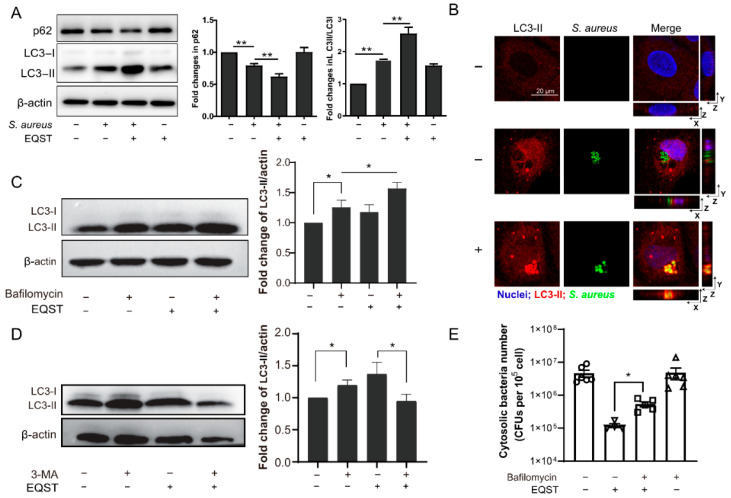
Equisetin-induced autophagy is associated with its antimicrobial activity. (**A**) The expressions of two marker proteins in autophagy (LC3 and p62) in IEC-6 cells. *S. aureus*-infected IEC-6 cells were incubated with 4 μg/mL of equisetin or in a complete medium for 4 h. All proteins were normalized to the level of β-actin. (**B**) Equisetin induced LC3 recruitment to the *S. aureus*-containing compartment. Images were taken by confocal microscopy. Bar: 20 μm. (**C**,**D**) The expression of LC3 protein in the IEC-6 cell-infected model, based on Western blot analysis. Cells were preincubated for bafilomycin (**C**) or 3-MA (**D**) for 2 h. All proteins were normalized to the level of β-actin. (**E**) Quantification of the number of intracellular *S. aureus*. Representative data are indicated as circles. After *S. aureus* infection, IEC-6 cells were co-cultured with gentamycin for 30 min to eliminate extracellular bacteria and then treated with equisetin. Data are representative of three independent experiments in (**A**–**D**) and six independent experiments in (**E**), and values are expressed in mean ± SD. * *p* < 0.05, ** *p* < 0.01, *p*-values were determined by unpaired t-tests, using PRISM 8.0.

As mentioned above, intracellular *S. aureus* manipulates autophagosome formation to escape autophagy (an important defense mechanism against intracellular pathogens) [24]. Therefore, we suspected that equisetin activates the autophagy pathway in host cells to eradicate the invasive pathogen. To tap into the role of autophagy in the interaction between host cells and equisetin, we dissected the expression levels of autophagy-related proteins LC3 (microtubule-associated protein light chain 3) and p62. During the autophagosome maturation, the cytoplasmic protein LC3-I is cleaved and recruited to generate LC3-II via specific lipidation after direct binding to the specific substrate protein p62 [25]. The level of the LC3-II is directly related to the amount of autophagosome, while the total p62 level is inversely correlated with the autophagy flux [26]. The immunofluorescence results exhibit that equisetin boosted the autophagy by increasing the ratio of LC3-II/LC3-I and reducing the level of p62 (Figure 2A). Compared with the vehicle group, equisetin treatment significantly promoted the LC3-II expression and decreased the number of intracellular bacteria (Figure 2B). However, the increase of autophagosomes (the increasing LC3-II) may also be due to the impediment of autophagic flux. To test whether the lysosomal function is inhibited by equisetin, we determined the expression of LC3 after a co-culture of the autophagy-inhibitor, bafilomycin [27]. Bafilomycin is a specific vacuolar V-ATPase-inhibitor that prevents the maturation of the autophagic vacuoles to disrupt the autophagy flux [28]. We found the levels of LC3-II elevated in the presence of equisetin plus bafilomycin, compared to the solo equisetin or bafilomycin treatments, indicating that equisetin indeed elicits the autophagic flux (Figure 2C).

To further characterize the role of equisetin in autophagy, we used 3-methyladenine (3-MA) to block the initiation part of autophagy-type III phosphatidylinositol 3-kinases (PI-3K) pathway [29]. The inhibition of the phagophore nucleation process successfully decreased the expression LC3, suggesting that equisetin had an effect on triggering autophagy. To deeper the underlying mechnism between autophagy activation and the elimination of intracellular bacteria clearance, we measured the population of internalized *S. aureus* treated with the autophagy inhibitor, bafilomycin. As shown in Figure 2E, the number of intracellular bacteria increased after co-culturing with bafilomycin and equisetin. These results confirm that equisetin potentiates autophagy to eliminate the intracellular *S. aureus*.

### 2.3. Equisetin Restricts Intracellular S. aureus Growth by Inducing Mitochondrial ROS Accumulaition

**Figure 3:**
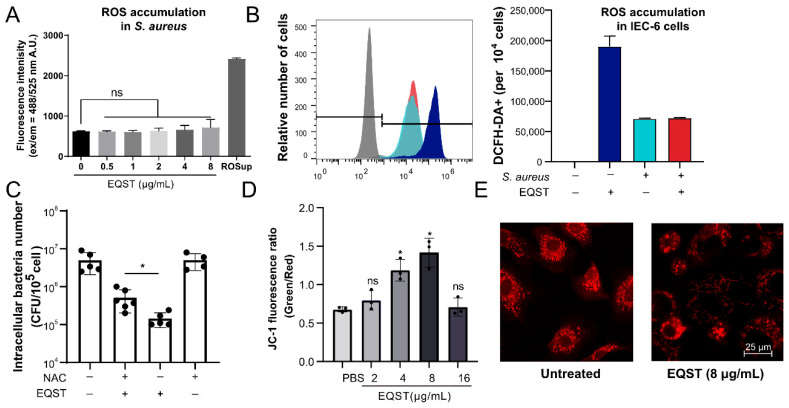
Equisetin increases cellular ROS accumulation through mitochondrial function mediation. (**A**) ROS content of *S. aureus* cells was stained by DCFH-DA before being exposed to equisetin. The fluorescence of DCFH-DA^+^ was detected by microplate reader. A.U. stands for absorbance unit. (**B**) ROS content of *S. aureus*-infected IEC-6 cells were stained with DCFH-DA before being co-incubation with equisetin. The fluorescence of DCFH-DA^+^ was detected by flow cytometer. *S. aureus* or EQST treatment was indicated as “+” or “ − ”. (**C**) The bacterial number of intracellular *S. aureus* in IEC-6 cells was analyzed by plate-counting assay. Representative data are indicated as circles. (**D**) The mitochondrial membrane potential of IEC-6 cells was labeled by JC-1 fluorescent probe. The fluorescence of JC-1 was analyzed by microplate reader. Representative data are indicated as circles. (**E**) Representative images of mitochondria in IEC-6 cells. Mitochondria were stained by MitoTracker-Red. Bar: 25 μm. Data are representative of three independent experiments in (**A**–**D**), and values are expressed in mean ± SD. * *p* < 0.05, *p*-values were determined by unpaired t-tests, using PRISM 8.0.

In the present study, we found that the suppression of bactericidal ability of equisetin was not completely recovered by specific autophagy inhibitor (Figure 2D). One potential explanation is that equisetin shows additional antibacterial strategies. Previous studies demonstrate that compounds with similar structures to equisetin perturb the mitochondrial membrane function and generate reactive oxygen species [30,31]. Therefore, we speculated that equisetin utilizes a similar way to eliminate intracellular *S. aureus*. To verify this hypothesis, the equisetin treatment increased ROS accumulation of IEC-6 cells, while the ROS content did not increase after exposure to the *S. aureus* in the broth medium (Figure 3A,B), indicating that ROS generation induced by equisetin requires mammalian cells. Correspondingly, cellular ROS accumulation increased in IEC-6 cells exposed to equisetin (Figure 3B). In subsequent studies, we evaluated the effect of ROS generation on bactericidal ability by adding the ROS scavenger, *N*-acetyl cysteine (NAC). The equisetin-mediated growth was inhibited by NAC (Figure 3C). Therefore, we inferred that ROS may play a critical role in the equisetin-mediated clearance of *S. aureus*.

ROS are mainly generated during mitochondrial respiratory activities and other cellular oxidases [26]. We speculated that equisetin-induced ROS generation is associated with a mitochondrial alteration. To verify this notion, we investigated the membrane potential (ΔΨm) of mitochondria in IEC-6 cells, based on a cyanine dye (JC-1) staining assay. The increase of green/red fluorescence intensity ratios indicated the mitochondrial potential decreased in a dose-dependent manner (Figure 3D). After the treatment with equisetin, the mitochondrial structure exhited short ministacks, which were neither aligned to each other nor laterally linked (Figure 3E). These results indicate that equisetin converts the mitochondrial structure, suggesting that equisetin modulates mitochondrial function resulting in ROS accumulation. Collectively, these results indicate that equisetin alters the mitochondrial membrane’s potential to mediate ROS generation potentiating invasive bacteria clearance.

### 2.4. Equisetin Shows the Potential to Control Intracellular S. aureus In Vivo

**Figure 4:**
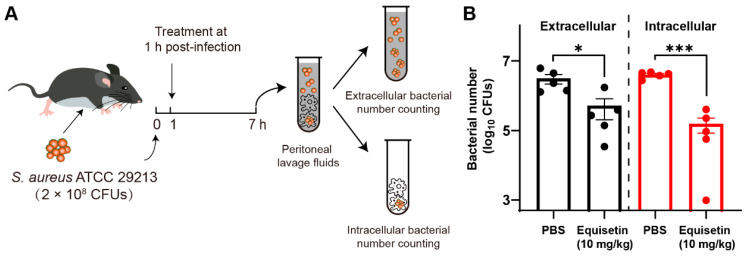
Equisetin reduces intracellular *S. aureus* in vivo. (**A**) Scheme of experimental protocol for the intracellular *S. aureus* infected mouse model. (**B**) The number of extracellular and intracellular *S. aureus* in peritoneal lavage fluids. Data are representative of five independent experiments and values are expressed in mean ± SEM. * *p* < 0.05, *** *p* < 0.001, *p*-values were determined by unpaired t-tests, using PRISM 8.0.

To evaluate the efficacy of equisetin in vivo, we established a peritoneal infection mouse model was established (Figure 4A): The mice were peritoneally treated with equisetin or PBS (as a negative control) after exposure to *S. aureus* ATCC 29213 for 1 h. The bacterial burden in each group was determined after 6 h. Consistent with the in vitro experiment results, equisetin effectively eliminated the *S. aureus* in vivo (Figure 4B) and protected the peritoneal cells from intracellular bacterial infection.

## 3. Discussion

*S. aureus* develops different strategies to evade or even hijack autophagy to escape autophagic response and replicate in host cells [32]. Our findings in *S. aureus*-infected IEC-6 cells are consistent with the previous reports that *S. aureus* inhibits autophagosome maturation and the fusion with lysosomes to thrive in host cells. The antimicrobial property of equisetin is associated with the activation of the autophagy. Firstly, it is noteworthy that the autophagy flux was activated by the equisetin in host cells. We estimated increased LC3Ⅱ/LC3Ⅰlevel with the addition of bafilomycin, which inhibited the downstream signaling pathway of autophagy(Figure 2A–D). It strongly suggests that equisetin treatment did not block the lysosome degradation to accumulate LC3-II. Secondly, IEC-6 cells treated with equisetin showed increased internalized bacterial loads after co-incubation with an autophagy inhibitor (Figure 2E), confirming that the equisetin-activated autophagy is essential for the antimicrobial activity against intracellular *S. aureus* infection.

Since the inhibition of autophagy did not completely abolish the antibacterial ability of equisetin, we speculated another host regulation pathway may be involved. Consistent with previous study, equisetin affected the mitochondrial membrane of the host cell (Figure 3E) [31,32]. Generally, the mitochondrial electron-transport chain is a major endogenous source of reactive oxygen species (ROS) by its oxidative metabolism and ATP synthesis function [26,33]. ROS are potent bactericidal adjuvants that can damage bacterial DNA, RNA, peptides, and lipids, resulting in bacterial cell death [34,35]. Equisetin induced mitochondrial membrane morphology change and mitochondrial membrane potential depolarization of host cells (Figure 3D,E), along with the increasing ROS content (Figure 3B). Considering that equisetin cannot promote the bacterial ROS, we concluded that equisetin accumulate host ROS production to reduce the intracellular bacteria (Figure 3A,B). Thus, mitochondrial-mediated ROS-promotion is another strategy of equisetin to restrict intracellular bacteria.

We noticed that equisetin induced less ROS in the bacteria-infected cells than in healthy cells (Figure 3B). Mitophagy is a quality control mechanism, which selectively and autophagically degrades the ROS-producing mitochondria to protect cells from oxidative stress [36]. In this scenario, we inferred that equisetin can induce mitophagy in diseased cells, leading to decreased mitochondrial number, as well as declined ROS relative content. Ubiquitination has been reported to recruit the autophagosomal machinery both in mitochondria and bacteria: E3 ligase Parkin ubiquitin acts on both mitochondria and surrounding phagosome of the intracellular bacteria; adaptor proteins including p62 and NBR1 are attracted to mitochondria during mitophagy and to bacteria during xenophagy [37]. Equisetin may orchestrate the interaction of the xenophagy and mitophagy to manage to clear the internalized bacteria.

## 4. Materials and Methods

### 4.1. Ethics Statement

The animal experiments were performed in accordance with the Administration of Affairs Concerning Experimental Animals Approved by the State Council of People’s Republic of China, and the relevant guidelines and regulations (ID: SKLAB-B-2010−003). The laboratory animal usage license is SYXK-2016-0008, certified by the Beijing Association for Science and Technology.

### 4.2. The Separation and Isolation of Equisetin

We isolated the fungus *Fusarium equiseti* 33-10 from deep-sea sponges, according to our previous work [16,38]. After cultivation, we extracted the fermentation by dissolving the sliced culture medium with ethyl acetate under ultrasound. Then, we redissolved it with 70% methyl alcohol and concentrated the fungal secondary metabolite. We isolated the active component of the supernatant, i.e., equisetin, by gradient eluting using HPLC. The structure of the equisetin was then identified by the HRESIMS and 1D, 2D NMR data mapping its spectroscopic data. Equisetin was diluted into DMSO in a concentration of 20 mg/mL, stored at −20 °C.

### 4.3. IEC-6 Cell Culture

The rat small intestine epithelial cells, IEC-6, from the ATCC (CRL-1592) were grown in Dulbecco’s Modified Eagle Medium (DMEM, Wisent Bioproducts, Nanjing, China), supplemented with 1% (*w*/*v*) heat-inactivated fetal bovine serum (Invitrogen, Waltham, MA, USA) and 1% penicillin–streptomycin. Cells were incubated in cultural flasks until sub-confluent (~80%). For testing, the IEC-6 cells were seeded at concentrations of 2 × 10^5^ cells/mL in 6-(2 mL), 12-(1 mL), or 24-well (0.5 mL) plates, and the experiments were started after 24 h.

### 4.4. Bacteria Incubation

*S. aureus* ATCC 29213 were grown in brain heart infusion broth (BHI, Land Bridge Technology, Beijing, China) or on BHI agar plates, at 37 °C. Vancomycin was obtained from the China Institute of Veterinary Drug Control.

### 4.5. Mouse Peritoneal Infection Model

The model mice were 6–8 week old male C57B/6 mice, randomly housed in a clean pathogen-free environment for one week before experiment. The in vivo efficacy of equisetin against *S. aureus* ATCC 29213 was assessed and peritoneal exudate cells were collected according to a previous protocol [39]. Briefly, three groups of C57B/6 mice (*n* = 5) were infected intraperitoneally with *S. aureus* ATCC 29213(2 × 10^8^ CFUs). Mice were intraperitoneally given 100 μL of the vehicle (PBS) and equisetin (10 mg/kg) at 1 h after infection via an intraperitoneal injection. After 6 h of infection, mice were humanely euthanized by CO_2_ asphyxiation, and then the peritoneal exudated cells were harvested with replicated injection and aspiration of cold harvest medium from peritoneum. The peritoneal exudated cells were centrifuged for 10 min at 400× *g*, 4 °C. The supernatant part of bacteria was diluted and enumerated by plate-counting on MH agar. The cell pellet was resuspended and incubated in a cold DEME supplied with gentamycin to remove the extracellular bacteria, and the cell counts were determined using hemacytometer. Serial dilutions of each suspension were plated on MH agar after removing the gentamycin medium for the enumeration of the bacterial colonies. The intracellular number was normalized by the volume of the peritoneal fluid.

### 4.6. Internalized Bacteria Analysis

The enumeration of the intracellular bacteria followed the previous protocol [14]. IEC-6 cells (2 × 10^5^ cell/mL × 1 mL per well in a 12-well cell culture plate) were infected with the *S. aureus* ATCC 29213 at the MOI of 1 for 4 h, then the medium was discarded and cells were washed with PBS three times to remove the non-internalized bacteria. The cells were incubated with fresh DMEM containing 100 μg/mL gentamycin for 15 min and then washed with PBS three times to remove the adherent bacteria. The previous research evidenced that gentamycin has no effect on killing intracellular *S. aureus* ATCC 29213 under such conditions. Subsequently, cells were incubated with DEME in the presence of equisetin for 0.5, 1, 2, 4, and 6 h. Cells were treated with 0.5% Triton for 10 min, and then the number of internalized *S. aureus* was counted by the plate-counting method. NAC and bafilomycin were treated 2h before equisetin appliment.

### 4.7. Immunofluorescence Staining

The IEC-6 cells were plated (2 × 10^5^ cell/mL × 2 mL in a six-well cell plate) and infected with *S. aureus* ATCC 29213 for 4 h. The extracellular bacteria were removed by co-culturing with gentamycin for 15 mins, then cells were incubated with equisetin in concentration of 4 μg/mL or 8 μg/mL. The cells were treated with LC3 (*Ex* = 552 nm, *Em* = 565 nm) and DAPI (*Ex* = 405 nm, *Em* = 454 nm), afterwards. In another immunofluorescence staining essay, the cells were stained with F-actin (*Ex* = 552 nm, *Em* = 565 nm) and DAPI (*Ex* = 405 nm, *Em* = 454 nm). For the static images, the cellular samples were fixed and stained and then captured by a Leica SP8 confocal microscope (Lecia, Teaneck NJ, Germany). The mitochondria of IEC-6 were tracked by MitoTracker (*Ex* = 552 nm, *Em* = 600 nm). The levels of fluorescence intensity were quantified using previous method [40].

### 4.8. Antibiotic Accumulation Analysis

The accumulations of equisetin in IEC-6 cells and bacteria were analyzed based on an LC–MS/MS analysis. After 4 h post-infection of the *S. aureus* ATCC 29213, the IEC-6 cells (2 × 10^5^ cell/mL × 1 mL per well in a 12-well cell culture plate) were treated with fresh DEME containing gentamycin to eradicate the non-internalized bacteria. Subsequently, the cells within the bacteria were treated with equisetin for 4 h. The extracellular drug accumulation was determined by supernatant of cell culture medium, which was collected and then analyzed via LC–MS/MS. The intracellular drug accumulation was analyzed after treating with 0.5% Triton, and the intra-bacteria drug load was determined according to the previous report [41].

### 4.9. Western Blotting

Cells were cultured and treated, as above, and then the levels of LC3 (cell signaling, Danvers, MA, USA) and p62 (Sigma, Darmstadt, Germany ) were tested by a Western blotting assay (2 × 10^5^ cell/mL × 2 mL in a six-well cell plate). Bafilomycin A1 (100 mM, KeyGEN BioTECH, Nanjing, China) and 3-MA (5 mM) were used to treat the IEC-6 cells before equisetin application. All the proteins were normalized to the level of β-actin. The gray values of the protein bands were quantified by open-source software Image J V 1.8.0.

### 4.10. ROS Detection

ROS of IEC-6 cells were tracked by an ROS assay kit (Beyotime, Shanghai, China). The change in the ROS was observed by a microplate reader (Tecan, infinite M200, Switzerland) (*Ex* = 488 nm, *Em* = 525 nm) and flow cytometer.

### 4.11. Mitochondrial Membrane Potential Detection

The changes to the mitochondrial membrane potential (∆Ψm) were assessed using a Mitochondrial Membrane Potential Assay Kit with JC-1 (Beyotime). After the cells were treated, as mentioned before (2 × 10^5^ cell/mL × 1 mL per well in a 12-well cell culture plate), the supernatant was removed and the cells were treated with JC-1 dye for 20 min at 37 °C. Finally, the cells were washed with a JC-1 buffer solution twice before observation under a laser confocal fluorescence microscope.

### 4.12. Cytotoxicity Assay

The effects of the equisetin on the IEC-6 cell viability were assessed by using a WST-1 Cell Proliferation and Cytotoxicity Assay Kit (Beyotime).

### 4.13. Statistical Analysis

The values reported are expressed as the mean standard deviation (SD) or standard error of mean (SEM). The Graphpad Prism 8.0.2 software (San Diego, CA, USA) was used for the graph plotting. A value of *p* < 0.05 was considered significant and is indicated with asterisks: * *p* < 0.05, ** *p* < 0.01 and *** *p* < 0.001; ns not significant and is indicated with ns. The statistical significance of the differences between the groups was performed by using an unpaired t-testing method on the Prism 8.0 software (refer to the previous method) [42].

## 5. Conclusions

Intracellular bacterial pathogens are difficult to treat due to the poor intracellular diffusion and retention of clinical antimicrobial agents [41]. Host-acting antibacterial compounds become a novel potential strategy to eradicate intracellular bacteria [13]. In this study, we found a potential marine antibiotic, equisetin, could diminish intracellular *S. aureus* through potentiating the host defenses, both in vivo and in vitro. Our results indicated equisetin utilize a dual-effect mechanism, including effectively activating the autophagy and boosting ROS generation, whereby the mitochondrial mediation of the host cells is activated to target the intracellular *S. aureus*. In addition, equisetin exerts a powerful bactericidal efficacy against extracellular bacteria with low cytotoxicity. Collectively, equisetin shows tremendous potential as a host-acting candidate to replenish antibacterial arsenal.

## Data Availability

Not applicable.
